# Functional Connectivity of Pain-Mediated Affect Regulation in Borderline Personality Disorder

**DOI:** 10.1371/journal.pone.0033293

**Published:** 2012-03-12

**Authors:** Inga Niedtfeld, Peter Kirsch, Lars Schulze, Sabine C. Herpertz, Martin Bohus, Christian Schmahl

**Affiliations:** 1 Department of Psychosomatic Medicine, Central Institute of Mental Health Mannheim, Medical Faculty Mannheim/Heidelberg University, Heidelberg, Germany; 2 Department of Clinical Psychology, Central Institute of Mental Health Mannheim, Medical Faculty Mannheim/Heidelberg University, Heidelberg, Germany; 3 Clinical Psychology and Psychotherapy, Freie Universität Berlin, Berlin, Germany; 4 Department of General Psychiatry, Center of Psychosocial Medicine, Heidelberg University, Heidelberg, Germany; Bellvitge Biomedical Research Institute-IDIBELL, Spain

## Abstract

Affective instability and self-injurious behavior are important features of Borderline Personality Disorder. Whereas affective instability may be caused by a pattern of limbic hyperreactivity paired with dysfunctional prefrontal regulation mechanisms, painful stimulation was found to reduce affective arousal at the neural level, possibly underlying the soothing effect of pain in BPD.

We used psychophysiological interactions to analyze functional connectivity of (para-) limbic brain structures (i.e. amygdala, insula, anterior cingulate cortex) in Borderline Personality Disorder in response to painful stimulation. Therefore, we re-analyzed a dataset from 20 patients with Borderline Personality Disorder and 23 healthy controls who took part in an fMRI-task inducing negative (versus neutral) affect and subsequently applying heat pain (versus warmth perception).

Results suggest an enhanced negative coupling between limbic as well as paralimbic regions and prefrontal regions, specifically with the medial and dorsolateral prefrontal cortex, when patients experienced pain in addition to emotional arousing pictures. When neutral pictures were combined with painful heat sensation, we found positive connectivity in Borderline Personality Disorder between (para-)limbic brain areas and parts of the basal ganglia (lentiform nucleus, putamen), as well areas involved in self-referential processing (precuneus and posterior cingulate).

We found further evidence for alterations in the emotion regulation process in Borderline Personality Disorder, in the way that pain improves the inhibition of limbic activity by prefrontal areas. This study provides new insights in pain processing in BPD, including enhanced coupling of limbic structures and basal ganglia.

## Introduction

Disturbed affective responding and affective dysregulation are core symptoms of patients with Borderline Personality Disorder (BPD) [1], [2]. Patients experience frequent mood swings and more pronounced negative emotions in everyday life than healthy control subjects (HC) [3], [4]. At a neurobiological level [5], findings point to a conjunction of dysfunctional prefrontal regulation mechanisms [6]–[8] and limbic hyperarousal, as possible explanations for affective instability. More specifically, apart from hyperactivation in the amygdala [9], [10] and insula [11], [12], patients also showed deviations in the anterior cingulate cortex (ACC [13], [14]). Furthermore, recent fMRI studies showed altered brain activations in the PFC in BPD during reappraisal [14], [15]. Although the reduced activation of prefrontal networks appears to be linked to limbic hyperreactivity, studies investigating the functional connectivity of these brain networks in BPD are still rare [6]: New and colleagues investigated relative glucose metabolic rate and found reduced baseline connectivity between amygdala and the anterior prefrontal cortex (PFC) in BPD patients. They conclude that the disconnection between PFC and amygdala may explain the difficulties to regulate emotions in BPD.

In conjunction with frequent states of aversive tension, patients prevalently resort to self-injurious behavior (SIB, also termed as non-suicidal self injury or deliberate self-injury [16], [17]). SIB is known to correspond to affective dysregulation [18] and presumably serves to escape from aversive tension, emotions, thoughts, or somatic sensations [19]. Accordingly, recent studies in BPD emphasize the important role of SIB in the regulation of negative affect [20], [21], [22]. Furthermore, patients with BPD show a decreased sensitivity to painful sensory stimulation; this sensitivity is further reduced under high levels of emotional tension [23], [24]. At the neuronal level, self-inflicted pain was found to activate the dorsolateral prefrontal cortex (dlPFC) along with significant deactivation of the amygdala and the perigenual ACC [25].

In earlier studies, we proposed that painful stimuli might serve as a possibility to distract attention from emotional contents [26]. According to general emotion regulation research, cognitive evaluation of emotional stimuli results in activation of prefrontal regions and attenuated activation in limbic regions [27], [28]. Furthermore, functional connectivity of amygdala and the prefrontal cortex predicts the extent of attenuation of negative affect [29]. Moreover, negative affect can be attenuated by directing the attentional focus away from aspects of a situation [30], [31]. This strategy is known as attentional shift or distraction. This mechanism is based on the assumption of limited processing capacity, resulting in a competition for neural resources between external stimuli [32]. Cognitive distraction from emotional contents resulted in reduced amygdala activity [33] and enhanced involvement of the precentral gyrus, middle frontal gyrus, as well as clusters in the superior parietal cortex in HC [28]. Conversely, two recent studies suggest that during pain, less attentional resources are available for the processing of negative emotions, resulting in reduced negative affect [34], [35]. One of those studies [35] found that acute fear of spiders was reduced by pain, induced by the cold pressor test. The other study [34] demonstrated that painful stimuli lead to decreased negative affect, especially in students with high emotional reactivity. Therefore, SIB in BPD might accomplish an attentional shift away from uncontrollable states of emotional tension, possibly compensating a lack of prefrontal control mechanisms.

In a recent study, we aimed to investigate the effects of pain on affect regulation in BPD more directly [36]. We presented negative (versus neutral) visual stimuli and subsequently induced heat pain (versus warmth perception) with thermal stimuli. In line with previous findings [10], [14], our results demonstrated (para-)limbic (amygdala, insula, ACC) hyperreactivity in response to emotional pictures in patients with BPD, which was true for both negative and neutral pictures. However, there was limited evidence that pain plays an exclusive role in the emotion regulation process in BPD. Although we found a decline in amygdala activity over time, it was not specific to patients with BPD or to painful stimulation. Disentangling the potential causes of amygdala deactivation over time was not possible by examining stimulus-related brain activation. Potential mechanisms of amygdala deactivation included an attentional shift caused by sensory stimuli per se [34], [33], the automatic use of cognitive regulation strategies or (re-) appraisal [37], or habituation processes [38].

To further explore potential brain mechanisms underlying the limbic deactivation observed over time [36], we re-analyzed our findings of pain-mediated emotion regulation in BPD to investigate functional connectivity by means of psychophysiological interaction analyses (PPI). By doing that, we focused on the connectivity between the regions that we had identified to be involved during emotional processing, namely the amygdala, the insula and the perigenual as well as dorsal ACC, expecting to reveal potential neural mechanisms of the role of pain in affect regulation in BPD. If painful stimuli cause a regulation of limbic areas in BPD by means of an attentional shift, we would expect greater inhibitory coupling of limbic areas and regions implicated in emotion regulation [29], more specifically regions implicated in attentional control (precentral gyrus and superior parietal regions [28], [39]. If differences in the (re-) appraisal of painful stimuli lead to diminished limbic activity in BPD, there should be enhanced connectivity to dorsolateral [40], medial [41] or ventrolateral [42] prefrontal regions. In contrast, in HC we expect inhibitory coupling of the aforementioned regions implicated in emotion regulation with limbic areas in response to negative pictures only in the control condition without painful stimulation.

## Methods

The study was approved by the Ethics Board of the University of Heidelberg. We only included participants with full capacity to content. Capacity to consent was established during a clinical interview. Twenty-six healthy female participants (HC; aged 27.13 years, SD = 8.26) and 23 female patients meeting the DSM-IV [43] criteria for BPD (aged 30.50 years, SD = 8.30) participated in the study (Comorbid Disoders in the BPD group: lifetime major depressive disorder (9), posttraumatic stress disorder (5), other anxiety disorders (9), substance abuse lifetime (5), eating disorder (6)). Patients were recruited by advertisement on websites dealing with BPD; HC by newspaper advertisement.

General exclusion criteria were organic brain disease, history of skull- or brain-damage, pregnancy, substance abuse during the last year, substance dependency (lifetime), severe neurological illnesses, metal in the body, left-handedness, claustrophobia, psychotropic medication (in the last eight weeks). Data sets from three HC and three patients had to be excluded due to problems during scanning or poor data quality. Both groups did not differ in age (t_(41)_ = 1.331, *p* = .19).

Diagnostic assessments was accomplished by trained diagnosticians and included the International Personality Disorder Examination (IPDE [44], inter-rater reliability: κ = .77), and the Structured Interview for DSM-IV Axis-I (SCID-I [45], κ = .69).

Healthy participants were also rated with the same semi-structured interviews to exclude any psychiatric diseases or substance abuse. The average of BPD criteria in patients was 6.5 (SD = 1.26). All met the criterion of affective instability and engaged in SIB in their lifetime. The majority of the patients engaged in SIB during the last year (85%; M = 108.1 days, SD = 95.9), mostly by cutting (30%), burning (24%), or beating (24%). In the majority of cases (70%), patients reported analgesia during SIB. The most important reported reason for SIB was the reduction of inner tension. Each subject provided written informed consent after the procedures had been fully explained.

## Experiment

Imaging data were collected using a Siemens TRIO-3T MRI scanner (Siemens Medical Systems, Erlangen, Germany). A high-resolution anatomical scan was acquired for each participant using 3-D magnetization-prepared-rapid-acquisition-gradient-echo (T1-weighted contrast, voxel size 1×1×1 mm3) as an individual template for normalization of functional data. For fMRI scans, T2-weighted gradient echo-planar-imaging for measurement of BOLD signal (field of view = 210×210 mm, voxel size = 3×3×3 mm, echo time = 30 ms, TR = 2500 ms) with 35 contiguous 3 mm slices in a 64×64 matrix was used. The first five scans were discarded to minimize T1 equilibration effects.

Study protocol and task procedure have been described in detail elsewhere [36]. In brief, each trial of the event-related fMRI design consisted of a negative or neutral picture stimulus from the International Affective Pictures System (IAPS [46]), which was presented for 12 seconds, and a temperature stimulus. The temperature stimulus was presented after the onset of picture stimulus, and lasted from second 4 to 12. The temperature was either warm, but not painful (39° Celsius), or a painful stimulus individually adjusted to the 60%-level of the subjective pain scale, which was assessed prior to the start of the experiment. The individual painful temperature of patients with BPD was 47.4°C (SD = 0.79), and 45.9°C (SD = 1.29) for healthy controls (t_(41)_ = 4.56 *p*<.001, *d* = 1.43). The pictures shown to the study subjects were selected according to their normative rating results, which resulted in a sample of 32 pictures with negative valence (M = 1.70, SD = 0.27) and high arousal (M = 6.67, SD = 0.50), and 32 pictures with neutral valence (M = 6.26, SD = 0.95) and low arousal (M = 2.91, SD = 0.28). After each trial, subjects had to indicate their current arousal level using the self-assessment manikin (SAM [47]). The inter-trial interval (white cross on black screen) was jittered from 6 to 10 seconds.

To analyze functional imaging data, we used standard procedures implemented in the statistical parametric mapping software package (SPM8, Wellcome Department of Cognitive Neurology, London, UK). The EPI time series were corrected for slice timing, spatially realigned and unwarped to correct for head motion, and normalized onto the T1-scan, which was previously segmented (using voxel-based morphometry) and normalized to the standard template provided by SPM8, resampled to 3 mm3 voxels, and smoothed with a Gaussian kernel with a full width at half maximum (FWHM) of 9 mm. For the event-related design, the statistical analyses relied upon the general linear model to model effects of interest (activation during negative vs. neutral pictures combined with painfully hot vs. warm temperature stimuli) and are reported elsewhere [36].

**Figure 1:**
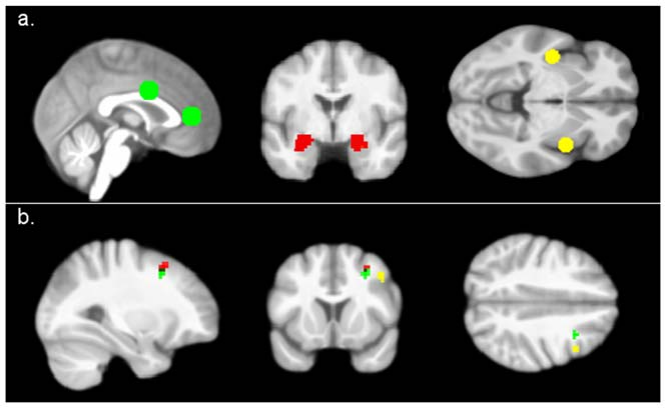
Seed Voxels of the PPI analyses. (1a); Prefrontal regions with negative coupling to the amygdala (red) insula (yellow) and perigenual ACC (green) in BPD when negative pictures were combined with painful temperature (1b).

In this study, we conducted analyses of psychophysiological interactions (PPI [48]) to investigate functional interactions between brain regions in relation to the experimental design. The PPI analysis is a method to assess task-sensitive changes in connectivity between brain regions. Thereby, it is possible to identify regions across the whole brain whose activity is more highly correlated with that of a seed region in one experimental condition than in another [48]. For the PPI-Analysis, we extracted the individual time course of activity from our regions of interest (insula, amygdala, and ACC) for every trial, which were subsequently used as seed region (see Figure 1a). We chose a data-driven approach to select the seed regions because we wanted to investigate the effect of pain on limbic areas processing negative affect. Therefore, we used a sphere of 9 mm around the local maxima of the whole-brain contrast (negative>neutral pictures) independent of group. Peak voxels were located in the left [−39, 3, −12] and the right [42, −9, 0] insula, as well as in the perigenual ACC [0, 42, 6] and dorsal ACC [0, 3, 30] (see [36]). Since the amygdala is a small structure, we used anatomical masks, defined by the Automated Anatomical Labeling software [49], smoothed with a full width at half maximum kernel of 9 mm, and thresholded with .10. The design matrix for the first level analysis contained the psychological regressor of the experimental paradigm, the time course of activation in the seed region, and the interaction of both. Separate first level analyses were computed for the contrasts negative painful>baseline, negative warm>baseline, neutral painful>baseline, and neutral warm>baseline as the psychological variables. In the second level analyses, the first level contrast of the interaction term (positive correlation of the seed region given the experimental condition) was used to compute separate full factorial designs for each structure and laterality. The second level full factorial design comprised the factors group (BPD vs. HC), valence (negative vs. neutral), and temperature (painful hot vs. warm). Additionally, we implemented covariates into the design matrix to control for the effects of objective temperature or subjective painfulness of sensory stimuli, as well as self-ratings of emotion regulation style (ERQ [50]). Clusters meeting a threshold of p<.001 (uncorrected) are presented; additionally we used a cluster extent correction procedure to compute the number of expected voxels per cluster according to random field theory [51].

## Results

The results for the psychometrics have been reported previously [36]. The following overview presents significant three-way (group by valence by temperature) interaction effects (see Table 1). All presented effects stayed significant when controlling for the covariates (objective temperature or subjective painfulness of sensory stimuli, self-ratings of emotion regulation style). For the complete results of the full factorial analyses, see Tables S1, S2, S3, S4, S5, S6.

**Table 1:** Full Factorial Analysis PPI, significant three-way interaction effects.

			k	p(FWE)	p(FDR)	p(unc)	quivZ		MNI	
Region and Effect	Brodmann Area	AAL						x	y	z
Left Amygdala: IE group×valence×temperature										
	Putamen	Lentiform Nucleus	14	0.768	0.87	0.000	3.644	24	−6	3
	BA 8	Middle Frontal Gyrus	11	0.782	0.87	0.000	3.63	30	15	48
Left Insula: IE group×valence×temperature										
	Putamen	Putamen	10	0.738	0.475	0.000	3.71	27	−9	−6
	BA 7	Precuneus	12	0.945	0.475	0.000	3.46	3	−60	30
Right Insula: IE group×valence×temperature										
		Caudate Nucleus	18	0.594	0.326	0.000	3.83	−15	3	12
	BA 31	Precuneus	17	0.668	0.326	0.000	3.77	12	−48	39
	BA 9	Middle Frontal Gyrus	13	0.786	0.326	0.000	3.67	42	12	42
Perigenual ACC: IE group×valence×temperature										
	BA 8	Middle Frontal Gyrus	8	0.774	1	0.000	3.67	30	12	42
Dorsal ACC: IE group×valence×temperature										
	Lateral Globus Pallidus	Lentiform Nucleus	14	0.54	0.72	0.000	3.859	−18	−3	9
	BA 8	Middle Frontal Gyrus	12	0.867	0.72	0.000	3.562	30	18	48

### Amygdala

**Figure 2:**
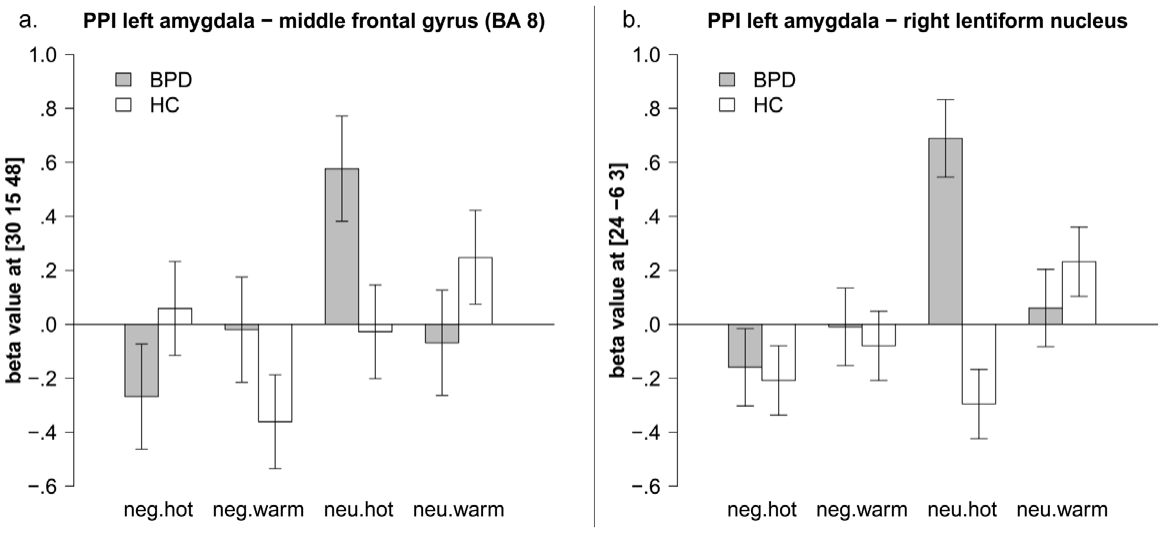
PPI full factorial analysis of positive correlation with the amygdala; interaction effects for group by valence by temperature, mean beta values and standard error of the mean of the peak voxels in the middle frontal gyrus (2a), and lentiform nucleus (2b).

The psychophysiological interaction analysis with the left amygdala revealed a group by valence by temperature interaction in the middle frontal gyrus (BA8) and the putamen (see Figure 2a and 2b, respectively). More specifically, HC showed a negative correlation of amygdala and middle frontal gyrus (BA8) only when negative pictures were combined with warm temperature, but no further coupling between the left amygdala and middle frontal gyrus was evident during the presentation of negative pictures with painful temperature. In contrast to HC, BPD patients showed a negative correlation of amygdala and middle frontal gyrus (BA8) only when negative pictures were combined with painfully hot temperature. In addition, patients with BPD showed a positive correlation between those neural structures when negative pictures were combined with warm temperature (see Figure 2a). Furthermore, the right putamen interacted more strongly with the left amygdala in BPD when neutral pictures were combined with painful temperature (see Figure 2b). The psychophysiological interaction analysis using the right amygdala as seed region revealed no significant three-way-interaction effects. For the complete results of the full factorial analysis, see Table S1 and S2.

### Insula

**Figure 3:**
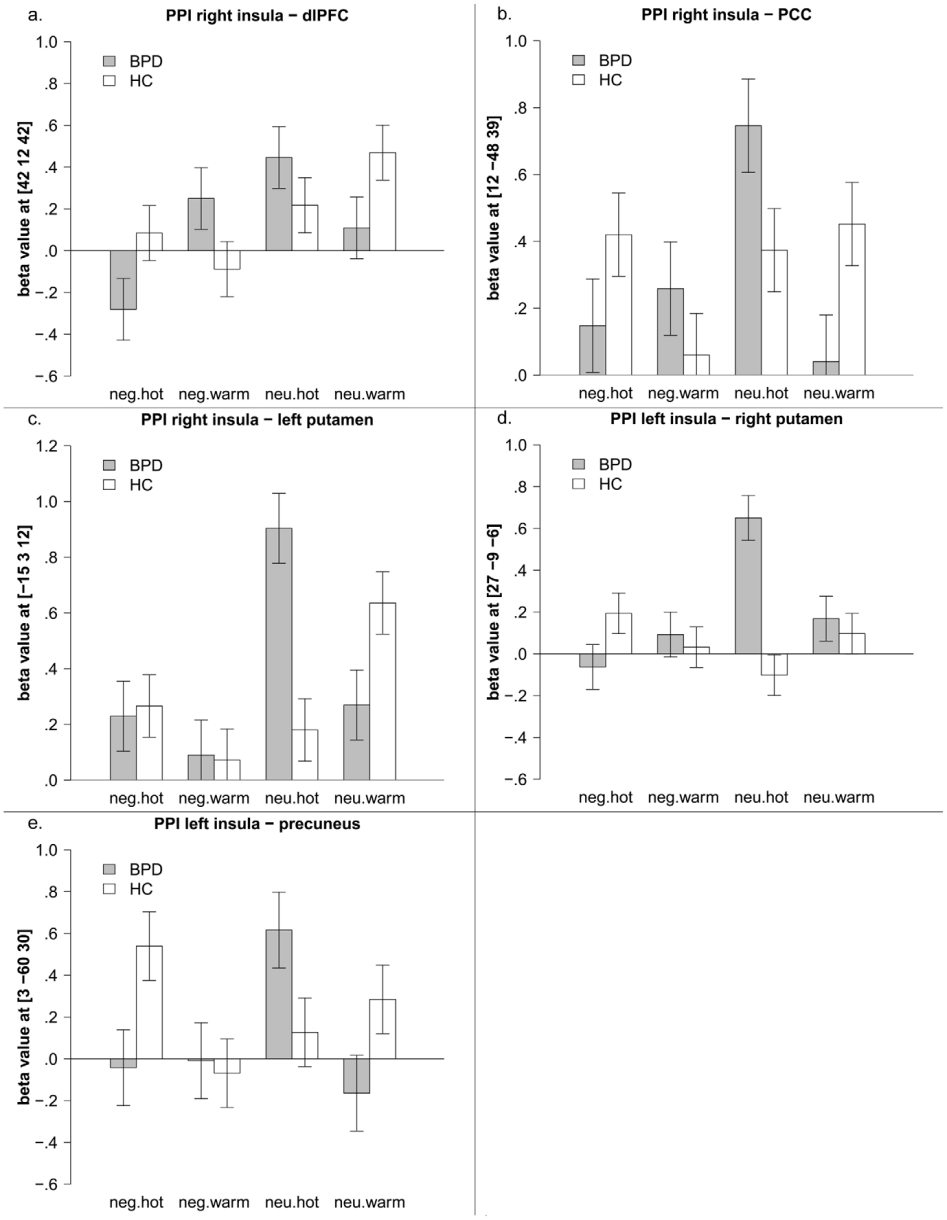
PPI full factorial analysis of positive correlation with the insula; interaction effect group by valence by temperature, mean beta values and standard error of the mean of the peak voxels in the putamen (3a), and precuneus (3b), dlPFC (3c), PCC (3d), nucleus caudatus (3e).

PPI analysis with the left insula as seed region illustrated a significant group by valence by temperature interaction for contralateral clusters in the right putamen and right precuneus (see Figure 3a and 3b, respectively). Both showed a positive correlation with the left insula in BPD when neutral pictures were combined with painful temperature, whereas a positive correlation was found in HC when negative pictures were paired with painful temperature.

Testing for connectivity with the right insula, a three-way interaction effect for group by valence by temperature revealed significant clusters in the left putamen, PCC, and dorsolateral prefrontal coretex (dlPFC, BA9). In line with the observed interaction of the left amygdala with middle frontal gyrus, patients with BPD showed a negative correlation of the right insula and the dlPFC only when negative pictures were combined with painful temperature (see Figure 3c). Conversely, patients show a positive correlation between those neural areas when negative pictures were combined with warm temperature. Regarding connectivity between right insula and dlPFC in HC, we found only weak correlations, which are in line with our results for the connectivity between amygdala and middle frontal gyrus.

Additionally, the positive correlation between the right insula and PCC was strongest in BPD when neutral pictures were combined with painful stimuli, while the interaction in HC was strongest when neutral pictures were combined with warm stimuli (see Figure 3d). For the putamen, BPD patients also showed a positive correlation when neutral pictures were combined with painful stimuli, and HC showed a correlation when warm stimuli were combined with neutral pictures (see Figure 3e). For the complete results of the full factorial analysis, see Table S3 and S4.

### ACC

**Figure 4:**
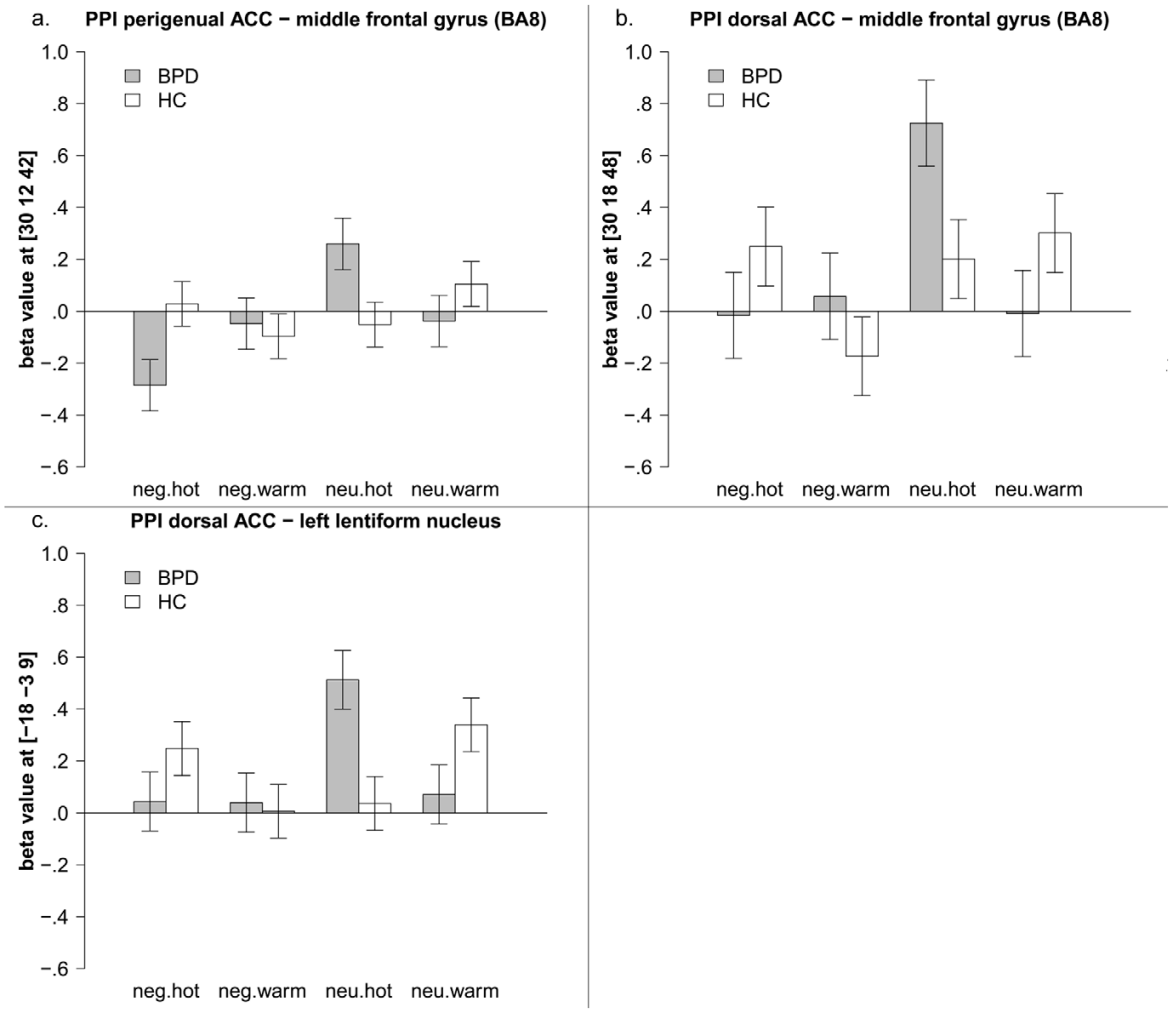
PPI full factorial analysis of positive interaction with the ACC, interaction effect group by valence by temperature, mean beta values and standard error of the mean of the peak voxels in the middle frontal gyrus (4a, 4b) and lentiform nucleus (4c).

Looking at correlations with the perigenual ACC, a group by valence by temperature interaction was observed for the middle frontal gyrus (BA8), showing a similar pattern as reported for the amygdala and insula before (see Figure 4a). Again, the correlation was negative in BPD patients when negative pictures were combined with painful temperature, whereas neutral pictures combined with warm stimuli resulted in a positive correlation. HC show the reverse pattern, i.e. a negative connectivity when negative pictures were combined with warm temperature, but a positive correlation for the condition negative pictures and painful temperature. For the complete results of the full factorial analysis, see Table S5.

PPI analysis of the dorsal ACC resulted in a three way interaction (group by valence by temperature) for the lentiform nucleus and the middle frontal gyrus (BA8). Both effects were mainly driven by a positive correlation in BPD patients when neutral pictures were combined with painful temperature (see Figure 4b and Figure 4c, respectively). For the complete results of the full factorial analysis, see Table S6.

## Discussion

The current analysis aimed to enhance our understanding of neural mechanisms underlying the role of pain in emotion regulation processes in BPD. Our results indicate that in BPD, negative co-variation of brain activity between (para-)limbic and prefrontal structures is only evident when patients experience physical pain during states of enhanced emotional reactivity. This was shown for the connection between the left amygdala and the middle frontal gyrus (BA8), the right insula and dlPFC (BA9), as well as for the coupling of perigenual ACC and middle frontal gyrus (BA8) (see Figure 1b). Thus, pain might result in increased inhibitory interactions (i.e. negative coupling) between neural areas associated with the processing of emotions and brain regions supporting the regulation of negative affect. More specifically, we found enhanced connectivity to prefrontal brain areas previously associated with the (re-) appraisal of stimuli (i.e. dlPFC [40], [41]) as well as attentional shift (i.e. middle frontal gyrus, [28]). Therefore, one could argue that pain in BPD results in enhanced inhibition of limbic regions by prefrontal control areas, and that this may be caused by two processes, on the one hand a different appraisal of painful stimuli in BPD and on the other hand attentional distraction by pain.

Assuming that pain causes increased inhibitory interactions in BPD allows us to address open questions regarding the effect of painful stimulation on affective arousal. In previous studies, we could not elucidate underlying causal mechanisms with custom statistical analyses, even though we found deactivation of the amygdala in response to pain [25], or identified a decrease in limbic activation over time [36]. By investigating functional connectivity with a PPI Analysis, we found that functional connectivity is altered in BPD specifically in response to pain, enabling inhibitory coupling with prefrontal control regions supporting emotion regulation [40], [41]. Conversely, HC showed this pattern of negative coupling between limbic and prefrontal regions only when negative pictures were combined with warm temperature. This inhibitory coupling within the control condition could be interpreted as a neural correlate of functional emotion regulation in HC [27], [29], although there was no sensory stimulation in the emotion regulation paradigms. Interestingly, there was no inhibitory coupling in HC when negative pictures were combined with painful stimulation, which could be further investigated in future research on emotion regulation in general.

Although earlier studies proposed an altered connectivity between limbic and prefrontal regions in BPD [15], [52], this is to our knowledge the first study directly addressing functional connectivity during an emotion regulation task in BPD. In addition to suggesting an altered coupling of emotion processing and emotion regulation structures in BPD, our results also provide additional evidence for alterations in the emotion regulation process by means of painful sensory stimulation [53], [36], [25]. Our findings support current theories on the function of self-injury [22] as a maladaptive strategy to regulate negative emotions [54]. Possible implications for psychotherapy of BPD can be deduced, accentuating the importance of distress tolerance strategies substituting dysfunctional attempts to diminish emotional tension. Additionally, it seems crucial to strengthen emotion regulation strategies, e.g. enhancing cognitive reappraisal. Both goals are targeted within Dialectical Behavior Therapy [55], teaching patients behavioral skills to handle states of emotional tension and to establish functional emotion regulation strategies.

Moreover, all limbic regions in BPD showed strong positive correlations to many other brain regions when neutral pictures were combined with hot temperature, which was not hypothesized a priori. Although the meaning of this correlation is not completely clear yet, one could tentatively interpret this positive coupling as further evidence for altered pain perception in BPD [25]. For instance, we found positive contralateral coupling between limbic regions and parts of the basal ganglia (lentiform nucleus, putamen) in BPD specifically for neutral pictures combined with painful temperature, whereas HC did not show this effect. This was found for the connection between left amygdala and right lentiform nucleus, left insula and right putamen right insula and left putamen, as well as for the dorsal ACC and lentiform nucleus. The putamen serves as the main input to the basal ganglia and receives afferents from many parts of the cortex [56]. Furthermore, the putamen was shown to have a central role in learning and memory by evaluating action-outcome contingencies. Therefore, our findings of higher correlations between limbic structures and basal ganglia when neutral pictures were combined with painful temperature may point to an enhanced processing of pain in terms of the anticipation of positive consequences. Painful stimuli were adapted individually and patients with BPD received higher temperatures to elicit the same moderate pain sensation as controls. Therefore, it was important to control statistically for these differences. Importantly, all reported effects stayed significant, so one can assume that they were not caused by differences in the objective temperature, nor by differences in subjective painfulness.

In addition, we found an enhanced positive connectivity in BPD between paralimbic brain areas and parts of the default mode network (precuneus and PCC) when neutral pictures were combined with painful temperature. The PCC is known to be a central node in the DMN of the brain, along with the precuneus [57]. Both are involved in conscious processing of information and self reflection [58]. Although the PCC was found to be deactivated during noxious thermal stimulation in HC [59], we found a positive correlation with the insula in BPD when neutral pictures were combined with painful stimuli. This enhanced connectivity of the insula and precuneus/PCC might reflect a disturbance of self-referential and emotional processing of pain in BPD [60]. The reported findings of enhanced connectivity between limbic structures and parts of the DMN seem to suggest that BPD experience pain as more purposive and more self-referential than HC [61].

However, our results need to be replicated and consolidated before it is possible to draw further conclusions. Although the analysis of psychophysiological interactions is an adequate method to detect connections between brain areas, it is based on correlations, and causal interpretations should be treated with caution. Future research should test explicit models of the assumed interactions, for example by Dynamic Causal Modeling (DCM), which requires a predefined network of interaction. Furthermore, twenty percent of the patients had a co-morbid PTSD, and it was shown that emotional reactivity is attenuated in BPD patients with PTSD [62]. Further research is needed to disentangle the role of pain in emotion regulation in this subgroup of patients.

In sum, examining the connectivity of brain networks in BPD has been proved to be a fruitful approach to shed light onto the neural processes underlying core self-injurious behavior. Healthy controls showed negative connectivity between prefrontal and limbic areas when negative pictures were combined with warm temperature. On the contrary, patients with BPD showed this negative connectivity only when negative pictures were combined with painful temperature. Therefore, one may conclude that painful stimuli result in improved regulatory processes in BPD. Furthermore, we suppose that differences in the appraisal of pain cause these differences, together with attentional distraction from emotional contents in response to pain. Our results provide evidence for an important role of pain in the emotion regulation process in BPD.

## Supporting Information

Table S1 **Full Factorial Analysis PPI, left Amygdala, all significant effects.**
(XLS)

Table S2 **Full Factorial Analysis PPI, right Amygdala, all significant effects.**
(XLS)

Table S3 **Full Factorial Analysis PPI, left Insula, all significant effects.**
(XLS)

Table S4 **Full Factorial Analysis PPI, right Insula, all significant effects.**
(XLS)

Table S5 **Full Factorial Analysis PPI, perigenual ACC, all significant effects.**
(XLS)

Table S6 **Full Factorial Analysis PPI, dorsal ACC, all significant effects.**
(XLS)
